# *Porphyromonas gingivalis* fimbriae

**DOI:** 10.3402/jom.v5i0.20265

**Published:** 2013-05-06

**Authors:** Morten Enersen, Kazuhiko Nakano, Atsuo Amano

**Affiliations:** 1 Institute of Oral Biology, Faculty of Dentistry, University of Oslo, Oslo, Norway; 2 Department of Pediatric Dentistry, Graduate School of Dentistry, Osaka University, Osaka, Japan; 3 Department of Preventive Dentistry, Graduate School of Dentistry, Osaka University, Osaka, Japan

**Keywords:** P. gingivalis, long fimbriae, short fimbriae, FimA, genotype, mfa1

## Abstract

Marginal periodontitis is not a homogeneous disease but is rather influenced by an intricate set of host susceptibility differences as well as diversities in virulence among the harbored organisms. It is likely that clonal heterogeneity of subpopulations with both high and low levels of pathogenicity exists among organisms harbored by individuals with negligible, slight, or even severe periodontal destruction. Therefore, specific virulent clones of periodontal pathogens may cause advanced and/or aggressive periodontitis. *Porphyromonas gingivalis* is a predominant periodontal pathogen that expresses a number of potential virulence factors involved in the pathogenesis of periodontitis, and accumulated evidence shows that its expression of heterogenic virulence properties is dependent on clonal diversity. Fimbriae are considered to be critical factors that mediate bacterial interactions with and invasion of host tissues, with *P. gingivalis* shown to express two distinct fimbria-molecules, long and short fimbriae, on the cell surface, both of which seem to be involved in development of periodontitis. Long fimbriae are classified into six types (I to V and Ib) based on the diversity of *fimA* genes encoding FimA (a subunit of long fimbriae). Studies of clones with type II *fimA* have revealed their significantly greater adhesive and invasive capabilities as compared to other *fimA* type clones. Long and short fimbriae induce various cytokine expressions such as IL-1α, IL-β, IL-6, and TNF-α, which result in alveolar bone resorption. Although the clonal diversity of short fimbriae is unclear, distinct short fimbria-molecules have been found in different strains. These fimbriae variations likely influence the development of periodontal disease.

*Porphyromonas gingivalis*, a Gram-negative black-pigmented anaerobic rod residing in subgingival biofilms, is widely recognized as a contributor to development of periodontal infections together with other oral pathogens (1–5). The species has also been reported to cause extraoral infections (6–9) and is suggested to play a role in the development of coronary heart disease, stroke, and diabetes mellitus, as well as preterm delivery of low birth-weight infants (7–13).

Although the central cause of periodontitis is loss of a healthy balance between microbial virulent agents and host immunity in host–parasite interactions, there are marked differences in progression rate and severity, as well as response to therapy in individuals affected by this infectious disorder (3, 5). Thus, periodontitis is not considered to be a homogeneous disease but is rather influenced by an intricate set of host susceptibility differences along with diversities in virulence among the harbored organisms. Indeed, *P. gingivalis* is present in periodontal pockets undergoing destruction as well as in healthy gingival margins (14, 15). Furthermore, clonal heterogeneity of subpopulations with both high and low levels of pathogenicity has been suggested to exist among periodontal pathogens harbored by individuals with negligible, slight, or even severe periodontal destruction. Therefore, specific virulent clones of the pathogens may be the cause of advanced and/or aggressive periodontitis.

*P. gingivalis* harbors an arsenal of virulence factors, including fimbriae, cysteine proteinases, hemagglutinins, and lipopolysaccharide (LPS), which along with its many interactions with the host immune system strongly support its potency as a pathogen (2, 16–18). Among those, fimbriae are a critical factor for colonization of *P. gingivalis* in subgingival regions, as they promote both bacterial adhesion to and invasion of targeted sites. Fimbriae variations and their effects on bacterial virulence are discussed below.

## Pili/fimbriae

Bacteria commonly express proteinaceous appendages on their outer surfaces. One class of extracellular polymers, known as pili or fimbriae (non-flagellar appendages), is used in attachment to and invasion of host cells, biofilm formation, cell motility, and transport of proteins and DNA across cell membranes. Pili and fimbriae are synonymous terms, with both commonly used, and are derived from Latin; pili for ‘hair’ or ‘fur’ and fimbriae for ‘fringe’. Since the first observations of these non-flagellar peritrichous appendages in the early 1950s, several distinct types of structures have been identified and characterized in Gram-negative bacteria, and later in Gram-positive bacteria (19–21).

Aggressive bacterial virulence factors that promote adherence and colonization of host organisms also include the well-studied protein adhesins, toxins, and translocated effector proteins. In all life forms, the general secretory pathway (GSP) (19, 20) provides a generic mechanism of protein transport across the cytoplasmic membrane. For secretion of proteins in Gram-positive species, the GSP may be sufficient, while Gram-negative bacteria have a more complex cell membrane and face the specific problem of management of protein transport across the outer membrane (OM) of their bacterial cells. The OM functions as a protective barrier against various antimicrobial host defenses, as well as a structure that enables the organisms to effectively colonize host cells and tissues. Therefore, Gram-negative bacteria have evolved special systems (20) for secretion and transport of proteins across the cell envelope.

Adhesins are a group of extracytoplasmic proteins found in pathogenic bacteria as well as environmental species, and they can be divided into two major classes. Fimbrial adhesins are composed of heteropolymers of pili subunits, while non-fimbrial adhesins consist of homotrimers or a single protein. Classification of the different types of pili/fimbriae has been established based on the different biosynthetic pathways of protein secretion and assembly (21).

The most studied fimbrial adhesins are type I pili [chaperone-usher (CU) pili], expressed by the Pap pili of uropathogenic *Escherichia* *coli*, and commensal *E. coli* isolates; type IV pili, expressed by the enteropathogenic *E. coli*, *Pseudomonas*, and *Neisseria* species, and curli pili, which are expressed by some strains of *E. coli*. In electron microscopic observations, type I pili have a peritrichous appearance on the cell surface, with rigid and rod-like structures 1–2 µm in length, while type IV pili are similar in length, though they appear to be more flexible (22), and curli pili have a coiled structure. All three types are formed by a non-covalent association of pilin subunits that comprise regular polymeric structures (20, 21). In addition, two other types have been described, type III secretion needle and type IV secretion pili (23).

## P. gingivalis fimbriae

*P. gingivalis* fimbriae seem to participate in nearly all interactions between the bacterium and the host, as well as with other bacteria (24). This pathogen expresses two distinct fimbria-molecules on its cell surface, one of which is composed of a subunit protein (named FimA or fimbrillin) encoded by the *fimA* gene, and termed long or long fimbriae, while the other consists of a subunit Mfa protein encoded by the *mfa1* gene and termed short, minor, or Mfa fimbriae (henceforth referred to as simply long and short fimbriae, respectively, in this review) (25). Short fimbriae are homopolymers of a subunit protein encoded by *mfa1*, with an apparent molecular mass of 75 kDa and antigenicity distinct from long fimbriae (26). Short fimbriae are shorter than long fimbriae and can only be visualized when the latter are absent. Both are apparently involved in the development of periodontitis (27).

## Long fimbriae

The fimbriae of *P. gingivalis* have been a focus of research in periodontal microbiology and pathogenesis for many years since Slots and Gibbons (28) first published their paper ‘*Bacteroides melaninogenicus* subsp. *asaccharolyticus* attachment to oral surfaces, and its possible role in colonization of the mouth and periodontal pockets’ in 1978. Earlier, bacterial surface components of other species that mediated attachment to host tissues were recognized as important pathogenic determinants (29). Electron microscopic observations of several black-pigmented anaerobic rods revealed that the organisms exhibited fine fibrillar appendages arranged in a peritrichous manner on the cell surface (30, 31). Some years later, Okuda and co-workers (31) noted that cell surface morphology and adherence to erythrocytes and human buccal epithelial cells vary among *Bacteroides* strains, including *B. gingivalis*. In negatively stained preparations, a dense network of fibers radiating from all dimensions of the cell surface of all strains examined was regularly observed. However, differential expression of fimbriae among strains of *P. gingivalis* has been reported (32).

Ultrastructural examinations of *P. gingivalis* strains have shown that peritrichous fimbriae vary in length from 0.3 to 3 µm and are 5 nm wide (2, 33). These structures have been classified as major or long fimbriae (FimA), based on their fimbrillin monomer composition, and range in size from 41 to 49 kDa (34–36). Even though ultrastructural studies were introduced 30 years ago, such examinations are still often used in combination with others for revealing additional information about this species (37). Various investigators have used purified fimbriae, recombinant fimbrillin, and antibodies to show that *P. gingivalis* fimbriae mediate bacterial adherence to a wide variety of molecules and oral substrates. These include salivary molecules, such as proline-rich proteins, proline-rich glycoproteins, statherins, oral epithelial cells, fibrinogen, fibronectin, and lactoferrin, and bacteria, such as oral streptococci and actinomyces species, which will be detailed (later in the article). Thus, long fimbriae are considered to be directly responsible for many of the adhesive properties of the organism, binding specifically to and activating various host cells, such as human epithelial, endothelial, and spleen cells, as well as peripheral blood monocytes, resulting in the release of inflammatory cytokines and several different adhesion molecules (4, 17, 27, 38).

## *fimA* genotypes and virulence

FimA is encoded by the *fimA* gene and occurs as a single copy in the chromosome of *P. gingivalis* (34, 39, 40). Based on its nucleotide sequence variation, the gene has been classified into six types (I, Ib, II, III, IV, V) (18, 39, 41, 42). That variation led to development of a PCR-based *fimA* genotyping method that is used for detection of possible relationships among the different genotypes, and virulence and disease. This method has been used in both experimental and clinical studies.

Nakagawa et al. (43) demonstrated that recombinant FimA protein corresponding to *fimA* genotype II has a greater ability to adhere to and invade human epithelial cells than FimA corresponding to the protein from other genotypes. The pathogenicity of the various *fimA* genotypes has also been evaluated in animal models, with *fimA* genotypes II, Ib, and IV shown to cause stronger infectious symptoms and inflammatory changes as compared to strains harboring *fimA* genotypes I and III (27, 44–46). On the other hand, Umeda et al. (47) found no significant difference between strains with different *fimA* genotypes in regard to adhesion to and invasion of epithelial KB cells. In addition, mutants in which the *fimA* type I gene was substituted with type II showed enhanced bacterial adhesion/invasion. In contrast, substitution of type II with type I resulted in diminished efficiency, supporting the notion that type II fimbriae are a critical determinant of virulence (48).

Results of several clinical studies also support findings that nucleotide variation of the gene is likely related to virulence. In chronic marginal periodontitis, *P. gingivalis* isolates with *fimA* genotypes II, IV, and Ib have been shown to be significantly more prevalent than isolates with other genotypes (27, 41, 42, 49–53). Also, studies of the pathogenic potential of distinct *fimA* genotypes in patients suffering from aggressive periodontitis have indicated that genotype II strains are more prevalent (54). In contrast, isolates with *fimA* genotype I are the most prevalent among *P. gingivalis-*positive healthy adults, followed by genotype V (52). In addition, *fimA* genotyping of cultured clinical strains of *P. gingivalis* sampled from individuals with periodontitis support previous findings that genotypes II, IV, and Ib are related to virulence (50).

A common observation among studies utilizing the *fimA* genotyping method is positive PCR cross reactivity to several different primer sets. Various explanations have been proposed, such as the presence of several different genotypes colonizing the same site (49, 51) and possible existence of new unidentified genotypes shown by the existence of *fimA* non-typable strains in some studies (41, 42, 49, 51, 53). However, genotyping of cultured *P. gingivalis* strains has detected only typable isolates and revealed similar findings as presented in clinical studies (50). Also, in endodontic infections, a *fimA* genotyping method resulted in similar findings as with strains from periodontal infections (55). To explain the observation of strains with multiple positive PCR reactions to several of the *fimA* primer sets, Enersen and co-workers (50) sequenced a selected number of isolates with new primers and then focused mainly on those showing multiple positive PCR reactions in a genotyping study. Their analysis verified a conserved *fimA* gene with only minor variations among the examined strains.

Multiple sequence alignments were presented by Enersen and co-workers (50), indicating that the genotyping method should be reevaluated partly due to the scant genetic variation between isolates within each genotype and between the different groups, in combination with the small differences in design of the primer sets. This was most important for detection of *fimA* I, Ib, and II. Their findings led to further development of new primers for detection of genotype II that was claimed to increase the accuracy of detection of the most prevalent genotype in diseased periodontal sites (56). A few studies suggested that other characteristics besides *fimA* gene variation may be responsible for the adhesion and invasion abilities (27, 47). That was supported by Inaba and co-workers (57), who reported heterogenic virulence among *fimA* genotype II strains, indicating that the variations in pathogenic potential and invasive efficiency were related to extracellular secreted gingipains, which will be discussed later.

In spite of the discrepancies related to the *fimA* genotyping method, a large number of experimental and clinical studies have indicated that some *fimA* genotypes may be important determinants of virulence for *P. gingivalis*. In addition, they may have a possible role in initiation and progression of cardiovascular diseases (see ‘Long fimbriae and cardiovascular diseases’ section).

## Protein structure of long fimbriae

The primary protein sequences of FimA share no significant homology with other described fimbrial proteins, indicating that *P. gingivalis* may possess a unique class of fimbria subunits (2, 36, 37, 39, 50).

The presence of an extremely long signal peptide, and requirements for Arg- and Lys-specific proteases (gingipains) for extracellular maturation, indicates that FimA is a novel group of fimbriae different from the type I and IV families (20). Shoji and co-workers (37) demonstrated that the major component proteins of the two cell surface structures, FimA and the 75-kDa protein related to short fimbriae, which are detailed later, seem to utilize the lipoprotein transport system with signal peptidase II, indicating a novel transport and assembly machinery with extracellular proteolytic polymerization.

In contrast, the major protein component of cell surface filaments of type I pili makes use of signal peptidase I for translocation across the cytoplasmic membrane, while type IV pili are dependent upon type IV-specific signal peptidases for their biogenesis.

The hypothesis and presented data supporting the variable virulence potential among different *fimA* genotypes of *P. gingivalis* imply a possible role for the tertiary structure in the function of FimA. However, the level of transcription of the gene is also an important factor (32).

Translation of *fimA* nucleotide sequences performed in a study by Enersen et al. (50) resulted in the same number of primary protein structures as sequence variants. Although the *fimA* gene was conserved, there were some minor variations between isolates belonging to the same genotype, resulting in corresponding variations in the primary protein sequence of the FimA monomer, which was also shown by Fujiwara and co-workers (39). Whether these mutations result in a FimA monomer with a changed structure that influences the pathogenicity of the isolate may partly depend on how the secondary structure of the molecule folds into a tertiary structure.

Presently, the tertiary structure of FimA is unknown, and an experimental structure resembling a protein with high homology to FimA has not been found (50, 57). Advanced bioinformatic data yet unpublished (58), based on results presented by Shoji and co-workers (37) as well as other bioinformatic sources, indicate that the structure of FimA may resemble the structure of protein NP_809975 of *Bacteroides thetaiotaomicron* found in the RCSB Protein Data Bank (structure 3GF8). From this structure, it is possible to speculate that fimbrillin generates multimers employing a ‘donor strand exchange’ in a manner resembling *E. coli* type I pili (59). FimA also appears to be hypervariable, which is consistent with its importance as a virulence factor for the species. Furthermore, the FimA sequence and subunits are changing rapidly over short evolutionary distances, making an experimental structure less useful, since the bacterium may be under positive selective pressure.

Based on alignments with similar sequences, it seems that conservation is highest in the signal sequence of *fimA* in *P. gingivalis* (58). This is in contrast to other bacteria in which the signal sequences have been found to be less conserved than the remaining part of the primary protein (58, 60), also indicating positive selection.

Although it has been claimed that FimA is unique due to lack of sequence homology between FimA and other fimbrial proteins, corresponding sequence alignments of fimbriae from other *Bacteroides* and parabacteroides species show a similarity to FimA, but the sequence homology may quickly become lost in this protein family due to extensive positive selection.

## Involvement of fimbriae in biofilm formation

Biofilm formation is a complex process involving reversible and irreversible bacterial attachment, microcolony formation, formation of a stable three-dimensional structure, and dispersion (61). Interspecies interactions help to develop the complex bacterial consortia in the gingival crevice. In the early phase, the initial bacterial colonizers (early colonizers), including streptococci, attach to available oral surfaces such as a salivary pellicle-coated tooth surface. Thereafter, later colonizers attach to the antecedent organisms and assemble into polymicrobial biofilm, which is mediated by co-adhesion (co-aggregation) with other bacterial species. *P. gingivalis* is able to aggregate with various oral Gram-positive and -negative species (62). Long fimbriae extend a significant distance from the bacterial cell wall, which suggests that they are the first bacterial components to interact with other bacteria as well as host cells. Indeed, *P. gingivalis* long fimbriae have been reported to mediate coadhesion with *Actinomyces viscosus* (63), *Treponema denticola* (64), *Streptococcus gordonii* (65), and *Streptococcus oralis* (66) via specific interactions with their receptors, including dentilisin of *T. denticola*, and glyceraldehyde-3-phosphate dehydrogenase (GAPDH) of *S. gordonii* and *S. oralis* (25). GAPDH is a well-characterized glycolytic protein involved in energy production and has been suggested to be a multi-functional house-keeping protein that is conserved by eubacteria and eukaryotic cells. The interaction between long fimbriae and GAPDH is the initial contact event that allows for localization of *P. gingivalis* on the streptococcal surface (67). The binding domains of the subunit protein FimA that mediate attachment to streptococci are localized to a C-terminal region spanning amino acid residues 266–337 (68). Human GAPDH has also been shown to bind to long fimbriae (69).

Short fimbriae reportedly mediate co-adhesion between *P. gingivalis* and *S. gordonii* via adhesin-receptor interactions with streptococcal SspA and SspB surface proteins (antigen I/II family) (61). SspA and B bind to short fimbriae, which increases binding avidity with a higher affinity than that of GAPDH to long fimbriae. Interestingly, following the development of a *P. gingivalis*–*S. gordonii* community, *mfa1* expression is down-regulated, presumably to alter the adhesin requirements of the antecedent organisms, as the streptococcal substrate becomes unavailable to later arriving *P. gingivalis* (70). The resulting phenotypic adaptation of *P. gingivalis* along with production of signaling molecules promote community development by recruitment of additional *P. gingivalis* cells from the planktonic phase (61).

The roles of long and short fimbriae in biofilm formation are likely different. The effects of a set of fimbriae as well as gingipains (Rgp and Kgp) were examined with regard to homotypic biofilm formation using deficient mutants (71). Those results suggested that long fimbriae promote initial biofilm formation and then exert a restraining regulation on biofilm maturation, whereas short fimbriae and Kgp have suppressive and regulatory roles during biofilm development. Furthermore, Rgp likely controls microcolony morphology and biovolume. Collectively, these molecules seem to act in a coordinated manner to regulate the development of mature biofilm.

*Streptococcus cristatus* is a later colonizer of tooth surfaces and attaches to *P. gingivalis* long fimbriae. However, arginine deiminase on the surface of *S. cristatus* initiates a signal transduction cascade in *P. gingivalis* that down-regulates *fimA* expression, resulting in fewer long fimbriae present on the *P. gingivalis* surface and no community formation between these microbes (72). Similarly, a secreted/excreted arginine deiminase from *Streptococcus intermedius* reportedly represses the expressions of *fimA* and *mfa1* (73), while it was also shown that this arginine deiminase prevents mono-species biofilm development by *P. gingivalis*, because *P. gingivalis* auto-aggregation is attributable to FimA protein (74, 75).

**Fig. 1:**
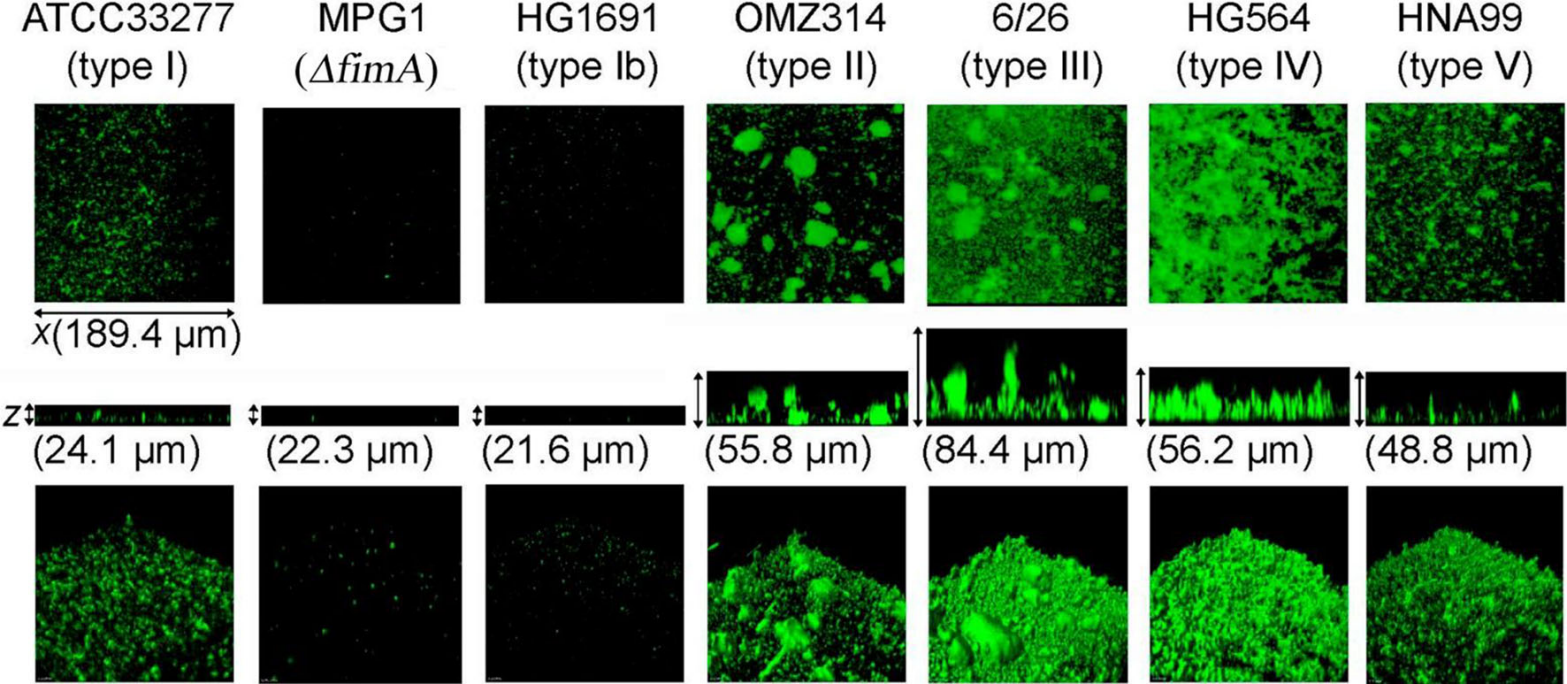
Homotypic biofilm formation by *P. gingivalis* strains with different types of long fimbriae and FimA (type I)-deficient mutant. *P. gingivalis* strains shown in the figure were stained with fluorescein (green). Biofilms developed on cover-glasses were observed with a confocal laser scanning microscope. Optical sections were obtained along the *z-*axis at 0.7-µm intervals, and three-dimensional images were reconstructed with imaging software. Data presented here were reproduced from Ref. 71, with permission.

Long fimbria variations interfere with the structures of biofilms formed by *P. gingivalis*. The effects of such variations on homotypic biofilm formation were examined using representative strains of each long fimbria type (I to V and Ib) (76). Biofilm structures formed by the six representative long fimbria type strains were apparently different (Fig. 1), and their characteristic features were confirmed to be closely related to long fimbria type in assays that utilized mutants with *fimA* substituted from type I to II and from type II to I.

## Induction of inflammatory responses by fimbriae

Toll-like receptors (TLRs) are a class of proteins that play a key role in the innate immune system. A previous study that employed a *P. gingivalis* mutant lacking long fimbriae identified reduced alveolar bone loss in a gnotobiotic rat model as compared with the wild type, while the inflammatory role of long fimbriae in atherosclerosis was also shown (77, 78). Since presentation of those findings, the effects of long fimbriae on immune responses have become well established (79).

TLRs co-cluster with CD14, CD36, CD55 (decay accelerating factor), complement receptor 3 (CR3; CD11b/CD18), CXC-chemokine receptor 4 (CXCR4), and growth differentiation factor 5 (GDF5) (80, 81). Long fimbriae have been shown to stimulate nuclear factor-κB (NF-κB) via TLR 2 and CD14, which results in induction of cytokines involved in bone resorption, such as tumor necrosis factor-α (TNF-α), interleukin-1β (IL-1β), IL-8, and IL-6 (80–84). Although CD14 is an essential co-receptor for activation of epithelial cells by long fimbriae (80), it was found that the cytokine responses (IL-6, IL-8, granulocyte-macrophage colony-stimulating factor, and TNF-α) of primary gingival epithelial cells to *P. gingivalis* are modest because of a lack of membrane-associated CD14 (85). In contrast, monocytes with CD14 respond vigorously to long fimbriae and secrete substantial quantities of IL-6, IL-8, and TNF-α (85). Long fimbriae also induce IL-1β, IL-8, MCP-1 (define), ICAM-1(vascular cell adhesion molecule-1), and E-selectin in human aortic endothelial cells (86). Although long fimbriae are only one of several inflammatory molecules of *P. gingivalis*, long fimbriae-deficient mutants showed significantly weaker cytokine responses as compared to wild-type strains (48). This is possibly due to the fact that long fimbriae are the most exterior components and likely the first to interact with host cell receptors, which is followed by initiation of intracellular signaling cascades.

Other studies have reported that short fimbriae strongly interacted with TLR2 and CD14, but not TLR4, and induced the expressions of cytokines, including IL-1α, IL-β, IL-6, and TNF-α, in both human monocyte cell lines and mouse macrophages (87, 88). In another study, *P. gingivalis* ATCC33277^T^ significantly induced periodontal bone loss in a mouse model, which was clearly suppressed by *mfa1* deletion in contrast to *fimA* deletion (74).

## Immune subversion mediated by long fimbriae

TLR signaling pathways crosstalk with the complement system, which is now recognized to exert functions above and beyond simple pathogen tagging and elimination (89). Recent evidence shows that *P. gingivalis* can suppress cell-mediated immunity by reducing the levels of interferon (IFN)-γ, another activator of cell-mediated immunity (90). *P. gingivalis* long fimbriae interact with CR3 to activate extracellular signal-regulated kinase 1/2 signaling, which inhibits IL-12 production mediated by TLR 2 signaling (91). In addition, the interaction of *P. gingivalis* or its purified fimbriae with CR3 inhibits the immune ability of LPS from other bacteria, such as *A*. *actinomycetemcomitans*, to induce IL-12 and IFN-γ in mouse macrophages or human monocytes. IL-12 is a key cytokine involved in pathogen clearance via its regulatory effects on the production of IFN-γ, a potent activator of macrophage microbicidal activity (92). Thus, CR3 activation, which signals cross talking with TLR 2 pathways and IL-12 inhibition, promotes the survival of *P. gingivali*s *in vitro* and *in vivo* (93).

Long fimbriae interact with CXCR4, a TLR2-associated receptor, which limits TLR2 activation in human monocytes and mouse macrophages. Furthermore, long fimbriae induce CXCR4-mediated activation of cAMP-dependent protein kinase A, which in turn inhibits TLR2-induced NF-κB activation in response to *P. gingivalis* (94). These results suggest that long fimbriae enable *P. gingivalis* to resist clearance *in vitro* and *in vivo*, promoting its adaptive fitness. In addition, Type II fimbriae from strain OMZ314 were shown to significantly induce cytokine expressions (48), thus the immune subversion potential may vary among *fimA* types, though definitive findings have not been presented.

## Long fimbriae and cardiovascular diseases

Long fimbria-deficient mutants were found to be relatively avirulent as compared to wild-type strains of *P. gingivalis* with regard to accelerating atherosclerotic plaque formation (79). Recently, oral-hematogenous spreading of *P. gingivalis* has received special attention for its possible association with several types of cardiovascular diseases. Several studies have reported detection of *P. gingivalis* in specimens collected from patients with cardiovascular diseases. In one of those, 42% of endarterectomy specimens showed a histological association of *P. gingivalis* with ulcer and thrombus formation (95), while another utilized a PCR assay method and found that 26% of carotid endarterectomy specimens were positive for the 16S rRNA fragment of *P. gingivalis* (96). Additional PCR analyses demonstrated that approximately 10% of studied heart valves and atheromatous plaque specimens were positive for *P. gingivalis* (97–99). On the other hand, Kozarov et al. (100) reported that the detection rate of *P. gingivalis* in atheromatous plaque specimens was approximately 90%, though they also noted that the rate for specimens taken from young patients was approximately 20%. Together, these results indicate localization of *P. gingivalis* in cardiovascular lesions, suggesting its association with development of cardiac diseases.

Long fimbriae were also shown to be associated with a necessary initial event in the development of atherogenesis by stimulating endothelial cell activation (101), while fimbria-mediated invasion was found to up-regulate the expressions of genes related to inflammation in aortic endothelial cells, leading to accelerated inflammatory responses directly in the aorta (102). The association of *fimA* genotype with virulence for cardiovascular diseases has been studied by several researchers. Pérez-Chaparro and co-workers (103) investigated seven blood isolates of *P. gingivalis* from dental patients who underwent scaling and root planing treatments, and showed that the *fimA* gentotypes of those isolated strains were composed of type IV (n=4), type II (n=2), and type III (n=1). As for the *fimA* genotypes of *P. gingivalis*-positive cardiovascular specimens, type IV was most frequently detected (45%), followed by type II (30%) (98). In addition, the abovementioned study demonstrated that approximately 50% of the dental biofilm specimens collected from cardiovascular patients were positive for *P. gingivalis*, with *fimA* type II found in 36%, type I in 29%, and type IV in 21%. These findings suggest that strains with specific *fimA* types, such as type II and IV, are associated with development of cardiovascular diseases. In fact, *fimA* type II, IV, and Ib strains were shown to cause more severe systemic inflammation in a mouse abscess model following subcutaneous injection as compared to those with other types (46). However, comparisons of the pathogenicity of strains with various *fimA* types to cardiovascular diseases in humans remain to be performed.

## Minor components of long fimbriae

Prototypical type I fimbriae of uropathogenic *E. coli* are structurally composed of a *fim* gene cluster encoding proteins, including the pilus shaft, adhesive tip components on the shaft, and proteins involved in the translocation of subunits across the cell envelope (25). After examining a typical model, the flanking region of the *P. gingivalis fimA* gene was comprehensively analyzed (104). The downstream ORFs, designated ORF 1, 2, 3, and 4, were found to encode 15-, 50-, 80-, and 19-kDa proteins, respectively. Among the specific antibodies against each of those proteins, two against the 50- and 80-kDa products reacted with purified fimbriae and were proposed to be minor components associated with fimbriae (105). Very recently, three ORFs (*fimC*, *fimD*, and *fimE*, named ORF 2, 3, and 4, respectively) were reported to encode minor components associated with FimA protein, and long fimbriae were suggested to comprise polymerized FimA and accessory proteins (FimCDE) encoded by genes of the fimbrial operon (105). FimE is known to be required for assembly of FimC and FimD onto FimA fibers (106), while the two genes upstream of *fimA* are involved in regulation of *fimA* expression under the control of the FimS–FimR two-component system (107, 108). In addition, *fimA* expression is controlled by expression levels of the FimA protein itself, as well as by Rgp and Kgp gingipains (109).

It was also reported that *fimC-*, *fimD-*, and *fimE*-deficient mutants lost their auto-aggregation ability, and long fimbriae purified from those mutants showed diminished efficiencies to bind to GAPDH of *S. oralis* as well as fibronectin and type I collagen (106). Thus, FimC, FimD, and FimE are adhesive tip components likely associated with long fimbriae, whereas recombinant FimA protein was demonstrated to express various binding activities, while it is also known to be an adhesive molecule (38). Another study showed that fimbriae of the *fimCDE* mutant lost their ability to down-regulate IL-12, a key cytokine involved in intracellular bacterial clearance, and induced a significantly lower level of alveolar bone loss as compared with the wild type (110). Furthermore, the mutant failed to exploit CXCR4 *in vivo* for immune subversion. Indeed, purified FimC and FimD (but not FimE) were shown to interact with CXCR4. In addition, FimC and FimD bound to fibronectin and type 1 collagen, whereas FimE failed to interact with these matrix proteins. Together, these reports indicate the importance of FimCDE in the virulence of *P. gingivalis* and assembly of fully functional fimbriae.

## Conclusions

Development of a useful genotyping testing tool for periodontal pathogens is necessary for therapeutic use. Future dentistry-related research will certainly produce such bacterial testing techniques for periodontal diagnosis, as well as medication and treatment strategies for affected individuals. However, additional efforts are required to investigate the exact relationship between genotypic variation and bacterial pathogenicity in periodontitis. Genomic variations of the long fimbria structures of *P. gingivalis* seem to be related to periodontitis initiation and progression. Furthermore, pan-genome analysis of *P. gingivalis* is expected to clarify the differences of virulence among strains. Future developments will be vital to identify the virulence/pathogenicity-related genes of *P. gingivalis*, while they will also be necessary for advancements in periodontal therapy and assessment of prognosis, by elucidating periodontal-related bacterial clones that contribute to disease.
